# Temporal integration characteristics of an image defined by binocular disparity cues

**DOI:** 10.1177/20416695231224138

**Published:** 2024-01-09

**Authors:** Fumiya Haraguchi, Rumi Hisakata, Hirohiko Kaneko

**Affiliations:** Department of Information and Communications Engineering, 13290 Tokyo Institute of Technology, Yokohama, Japan; Department of Information and Communications Engineering, 13290 Tokyo Institute of Technology, Yokohama, Japan; Department of Information and Communications Engineering, 13290 Tokyo Institute of Technology, Yokohama, Japan

**Keywords:** temporal integration, binocular disparity, shape recognition, slit vision

## Abstract

We can correctly recognize the content of an image by presenting all of the elements within a limited time, such as in a slit view or a divided painting image. It is important to clarify how temporally divided information is integrated and perceived to understand the temporal properties of the information-processing mechanism of visual systems. Previous studies related to this topic have often used two-dimensional pictorial stimuli; however, few have considered the temporal integration of binocular disparity for the recognition of objects defined with disparity. In this study, we examined image recognition properties based on the temporal integration of binocular disparity, by comparing that based on the temporal integration of luminance. The effect of element onset asynchrony (the time lag among presented elements) was somewhat similar between disparity and luminance with respect to randomly divided elements. On the other hand, under slit-vision conditions, the tolerance range of spatiotemporal integration for luminance stimuli was much wider than that for disparity stimuli. These results indicate that the temporal integration mechanism in localized areas is common to disparity and luminance, but that for global motion shows differences between the two mechanisms. Thus, we conclude that global motion has little contribution to the temporal integration of binocular disparity information for image recognition.

The visual system integrates information spatially and temporally in the visual field to perceive the outside world. One of the reasons for this property is that visual acuity differs greatly depending on the position in the visual field, given that the resolution is high in the fovea centralis but lower in the peripheral field. To correctly recognize the entire visual scene, it is necessary to integrate information presented with spatial and temporal differences due to eye movement.

Some studies have shown that it is possible to integrate visual information with time lags for scene recognition. Ikeda and Uchikawa (1978) investigated the mechanism of temporal integration for object recognition by measuring the lags when observing a painting divided into elements and displayed with temporal asynchronies. When all of the elements were presented within a short period, the content of the painting was recognized. By contrast, if it took a long time to present all of the elements, the participants were unable to recognize what was drawn. This temporal integration of visual patterns is explained by the temporal properties of lower-order visual processing, such as visual information storage, which retains visual information after it has disappeared (Coltheart, 1980; Nikolić et al., 2009; Teeuwen et al., 2021). It has been shown that patterns presented in a short time window of a few hundred milliseconds can be perceived as if all were seen simultaneously (Ikeda & Uchikawa, 1978). In the experiment by Ikeda and Uchikawa (1978), there were two conditions: one in which the participants were asked to verbally respond to patterns as image recognition, and the other in which they were asked to draw the patterns they observed. In the drawing condition, the participants were able to replay the image pattern even if they did not understand the meaning of the pattern, and they were able to understand the meaning of the pattern only when they saw the pattern replayed and drawn by themselves. This tendency was also common in the condition of tactile recognition of the image pattern formed from the unevenness of the plastic stimulus. It has also been shown that even if a pattern is not recognized as meaningful at the time of stimulus observation, it can be recognized because of replaying and drawing the pattern as a picture. Such multisensory and time-consuming pattern recognition is qualitatively different from the perceptual image obtained with a short time presentation. Although it has been suggested that higher-order working memory is involved in pattern recognition resulting from the integration of tactile and visual sensation (Matusz et al., 2017; Quak et al., 2015; Shioiri et al., 1983), the perceptual image obtained after a short presentation time of several hundred milliseconds is basically a function of the lower-order temporal integration of visual processing (e.g., Burr & Morrone, 1993). It can be regarded as a kind of bottom-up processing of perception.

On the other hand, it is known that even in the temporal integration of lower-order visual processing, different time windows can be obtained depending on the task and the way the visual stimuli are presented (Fink et al., 2006; Melcher et al., 2014). For many types of stimulus presentations, two stimuli presented within 40 ms are synchronously perceived; however, in some situations, they can be integrated over longer time windows. For example, even if a stimulus is presented behind a thin slit and only a small portion of the stimulus is visible, participants recognize the whole object behind the slit. In addition, Parks (1965) found that when a stimulus moves behind a narrow slit, the perception of the stimulus differs depending on its velocity (presentation duration of the stimulus); specifically, they showed that if the stimulus moves quickly behind the slit, the size is perceived as shrunk, whereas if it moves slowly, participants cannot perceive the whole object and the size appears to be expanded. This fact indicates that we can reconstruct information behind the slit from the spatiotemporal information of the motion of the slit window. Just as tactile reproduction (drawing) of a spatially randomly presented image can reproduce it (Ikeda & Uchikawa, 1978), slit viewing involve higher-order visual integration processing in which local structures given in fragments are supplemented by linking them together from global motion information. Such temporal completion may be used not only in the special situation of slit viewing, but also in the integration of scenes across saccadic eye movements.

Temporal integration of visual information should be done within reasonable temporal limits, but there is no guarantee or reason that each visual attribute will have similar temporal integration properties. For example, there are various cues to information about the depth of an object or scene. For example, what are the temporal integration properties of binocular disparity, which are obtained by integrating information from both eyes? Some studies have investigated three-dimensional (3D) object recognition from the stimulus movement, although most slit-vision studies have focused on two-dimensional pictorial recognition of the images. Research has shown that shape perception is possible, even in the slit vision of a rotating object or a cube frame (Norman et al., 2021). This indicates that even under slit-vision conditions, a participant is able to temporally integrate the local motion information to recognize and estimate the whole object's motion and restore the 3D shape. To date, studies of 3D-shaped slit vision have dealt mostly with the temporal integration of monocular depth cues, such as shading and rotational motion. We assume here that the temporal integration of binocular disparity is an important cue for detecting object depth in 3D shape recognition. However, no studies have investigated the temporal integration of object recognition defined by binocular disparity.

In this study, we attempted to clarify the properties of temporal integration processing of binocular disparity for shape recognition by comparing it to that of luminance information. We used two-digit numbers defined by binocular disparity or luminance as targets in the stimulus to measure the response accuracy, in which the numbers consisted of random dots that were divided into groups and presented with time lags.

In Experiment 1, the stimulus elements were divided into groups, and the groups were presented with element onset asynchrony (EOA), in which we manipulated the duration between the onset of the first presented elements and that of the last presented elements, such that the total presentation duration was variable. The image was defined by binocular disparity or luminance. We examined how the elements presented with a time lag were integrated and perceived. Our results showed that the larger the EOA between the element group, the more difficult it was to integrate the stimulus using either disparity or luminance for perceiving the image. In Experiment 1, the number of stimuli presented to the participant at the same time (hereinafter referred to as the instantaneous maximum ratio [IMR] of the stimulus element) changed with the EOA conditions. To clarify whether EOA or the IMR is important for recognizing the object's shape, we fixed the IMR among the conditions of the presentation durations in the next experiment. We found that even when the IMR was constant, the performance of image recognition in the random presentation decreased as the EOA among elements increased. Furthermore, the EOA effect did not differ between stimuli defined by disparity or luminance.

Furthermore, we examined the effect of the presentation order of elements in Experiment 2. We compared a slit-vision presentation, in which the stimulus elements were presented in order from one of the edges of the image (left or right), with that of a random presentation in which elements were presented in random order. Under the slit-vision condition, the rate of correct answers for the luminance stimulus increased rapidly with the EOA, but that for the disparity stimulus increased more slowly.

In Experiment 3, we investigated the effect of eye movement due to the movement of the slit window, in which we compared the conditions of slit motion and stimulus motion on the background behind a fixed-slit window. Under the condition of slit motion, the slit moved in front of a stationary image. Under stimulus motion, on the other hand, the stimulus image moved behind the stationary slit. In both conditions, the image sequences presented on the retina would be the same. We found that the rate of correct answers did not change much for either condition for the stimulus defined by binocular disparity, suggesting that the eye movement following the slit window did not affect the results of Experiment 2.

Taken together, the results of the three experiments suggest that the characteristics of local temporal integration for both disparity and luminance are similar, whereas those of spatiotemporal integration based on motion detection mechanisms differ. Thus, motion information does not appear to contribute to the integration of binocular disparity for shape perception.

## Experiment 1: Temporal Integration of Binocular Disparity and Luminance for Shape Recognition

In Experiment 1, two types of stimuli, one defined by luminance and the other defined by binocular disparity, were used to compare the properties of their temporal integration for image perception. The stimulus consisted of random dots and the task was to detect two-digit numbers defined by binocular disparity or luminance. We divided the stimulus into element groups and introduced EOA while measuring the recognition rate of the two-digit numbers.

### Methods

#### Participants

Eight participants with normal visual acuity or corrected normal visual acuity, including the three authors, participated in the experiment. We conducted a simple stereo test (Stereo Fly test, Stereo Optical Co., Chicago, IL, USA) to measure stereoscopic visual acuity, confirming that all participants could detect disparity at a minimum of 200 arcsec of visual angle. Notably, one participant in the preliminary experiment, who had a rate of correct answers of <90% in the recognition of stimuli using disparity, did not perform the main experiment. The experiment was conducted with the approval of the Tokyo Institute of Technology Ethics Review Board.

#### Apparatus

In the experiment, a haploscope consisting of a control computer (MacBook Pro, OS X Yosemite, Apple, Inc., Cupertino, CA, USA), two mirrors placed at right angles, and two displays placed in parallel (Display SONY Professional Video Monitor, PVM-A170; 60 fps; 36.58 × 20.57 cm^2^, viewing angle 53.2° × 31.5°; Sony Corp., Tokyo, Japan) was used. The experiment was conducted in a dark room. The observation distance from the eye to the center of the display was 36.5 cm.

#### Stimuli

**Figure 1.**
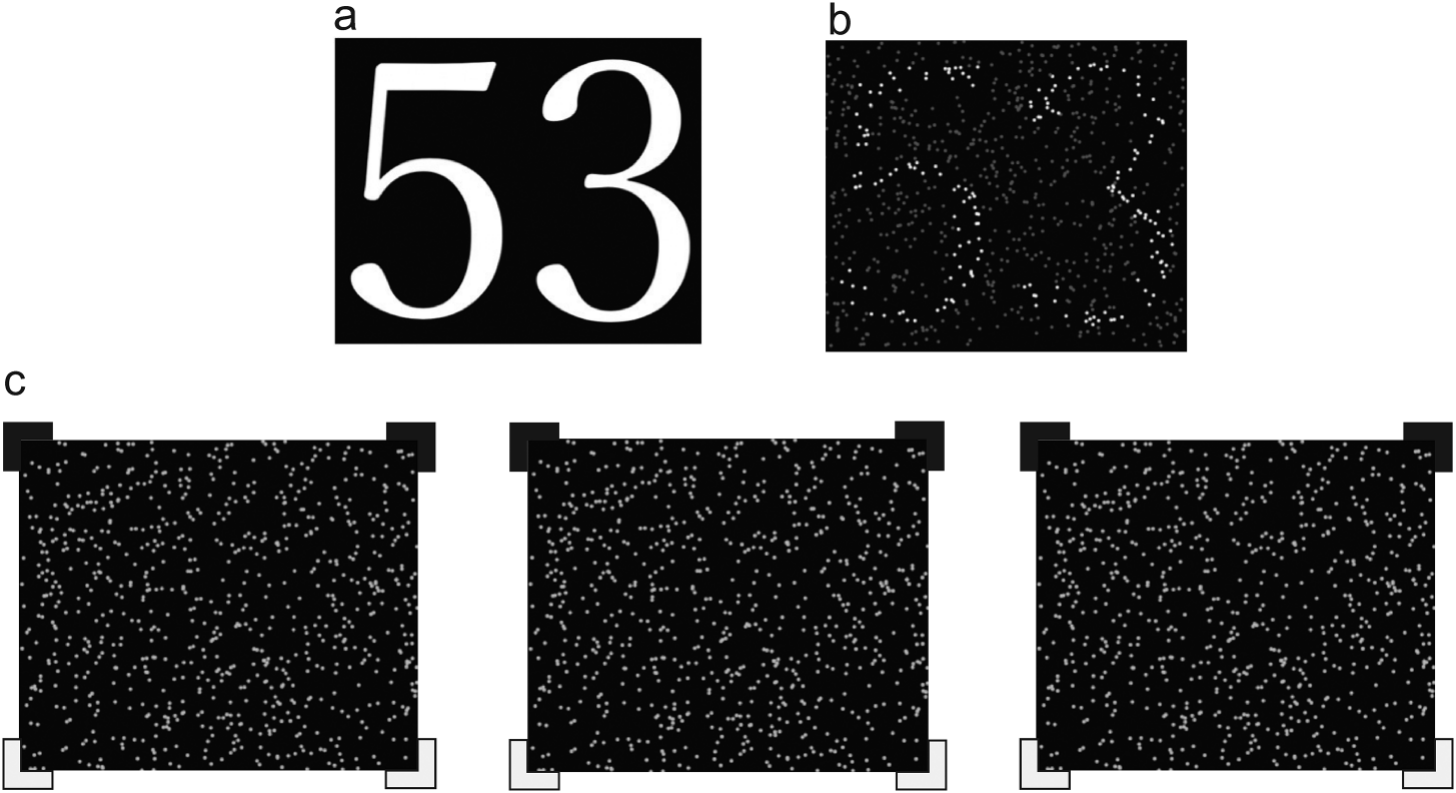
(a) The original image of a two-digit number. (b) The dot image defined by luminance difference. (c) The dot image defined by binocular disparity.

The stimulus consisted of random dots (3 pixels in diameter, 800 dots in total) within an area of 8.95° × 7.46°. In this random dot stimulus, a two-digit number was defined by binocular disparity or luminance. The participant's task was to identify the two-digit number. The numbers were randomly selected from eight types from 0 to 9, excluding “1” and “7” (Figure 1(a)). The reason for excluding “1” and “7” is that these numbers/digits are easier to recognize compared to the others. This resulted in a chance level of 1.56% for a double-digit percentage of correct answers. Each dot was presented for 300 ms.

#### Conditions

In the luminance condition, the numbers were presented by a group of dots with 80 cd/m^2^, and the other group dots were displayed using a lower luminance (Figure 1(b)). In the disparity condition, the numbers were presented by a dot group with a crossed disparity, which was determined individually in the preliminary experiment. The other dot groups were displayed with zero disparity with respect to the display surface (Figure 1(c)). The numbers defined by luminance could be recognized, even with a single eye. By contrast, the numbers defined by disparity were detected only with binocular vision. The method for determining the disparity magnitude and the luminance is described in the section below.

**Figure 2.**
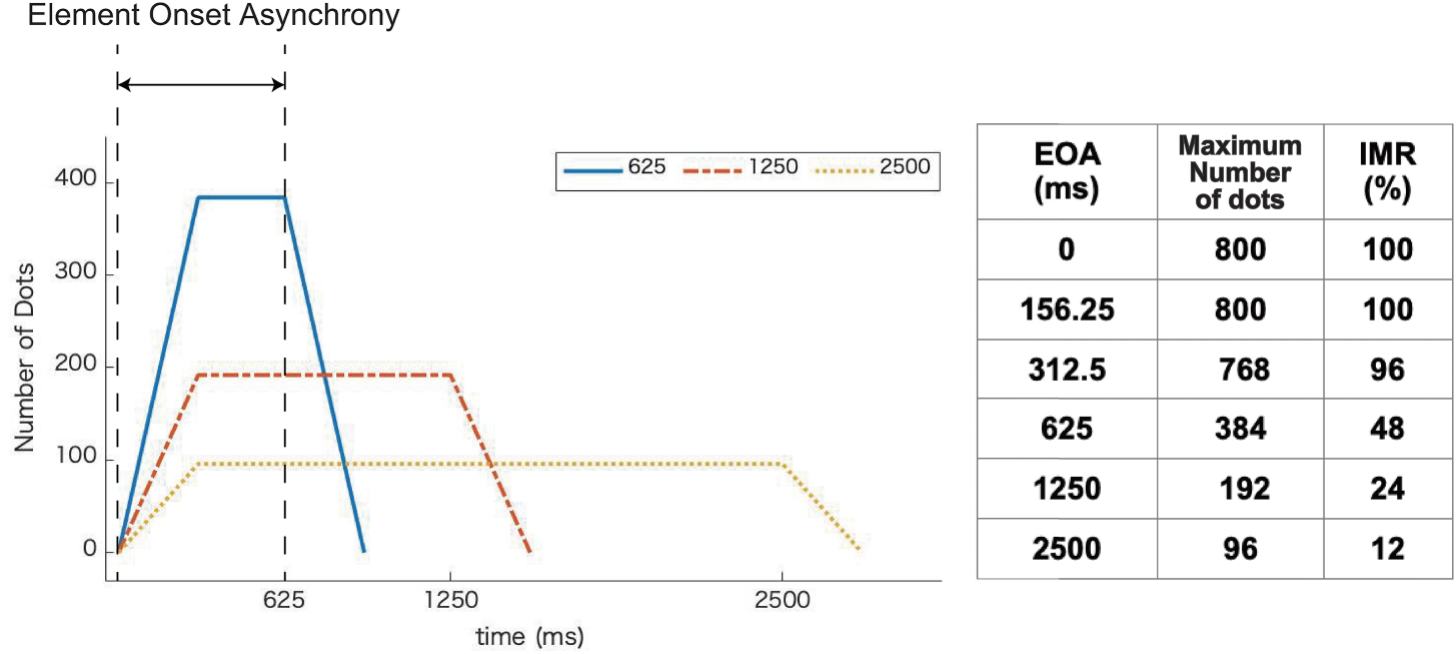
Time course of the number of presented dots in three element onset asynchrony (EOA) conditions (625, 1250, and 2500 ms). The horizontal axis is the time from the stimulus onset (ms) and the vertical axis is the number of presented dots.

The time from the presentation of the first dot to the presentation of the last dot was manipulated. We defined this value as the EOA, in which we tested six conditions: 0, 156.25, 312.5, 625, 1250, and 2500 ms. Figure 2 shows the time course of the number of dots presented for three of the EOA conditions (625, 1250, and 2500 ms). The maximum number of dots presented decreased as the EOA became longer, given that the presentation duration of each dot was constant (300 ms). When the presentation of the stimulus was initiated, the number of displayed dots increased gradually, reaching a specific value, followed by a gradual decrease. The table in Figure 2 shows the maximum number and percentage of dots presented at the same time for each EOA condition, specifically defined as the IMR, respectively, in this research. For example, if the IMR is 50%, this means that in one frame, an instantaneous maximum number of 400 dots was presented, which is up to half of the total of 800 dots.

#### Procedures

Prior to the main experiment, we conducted a preliminary experiment to compare the rate of correct answers for the stimuli with disparity and luminance for each participant. In the experiment, all of the stimulus elements were presented for 300 ms at the same time. This presentation was the same as the presentation condition of 0 ms lag used in the main experiment. In the preexperiment, the disparity or luminance was manipulated by the simple 1-up/1-down method to find the rate of correct answers of 90%. When the disparity or luminance exceeded the defined range (between −6 and 0 arcmin for disparity and between 0 and 50 cd/m^2^ for luminance) the stimulus remained at the maximum or minimum value. In total, 120 trials were conducted for each of the disparity and luminance stimuli. The results were fitted by the normal cumulative distribution function using the maximum likelihood method, and we calculated the luminance and disparity at which the rate of correct answers was 90% for each participant. These values were used in the main experiment.

In the main experiment, we randomly presented the EOA conditions within a block. Thirty trials were repeated for each EOA condition, and there were 12 blocks, giving a total of 360 trials. The participant fixated on the center point and pressed the keyboard to start the stimulus presentation. After the stimulus presentation, the participant responded by identifying the two numbers presented, using a numerical keyboard. The rate of correct answers for each condition was calculated based on the number of trials in which both digits were correct.

### Results

**Figure 3.**
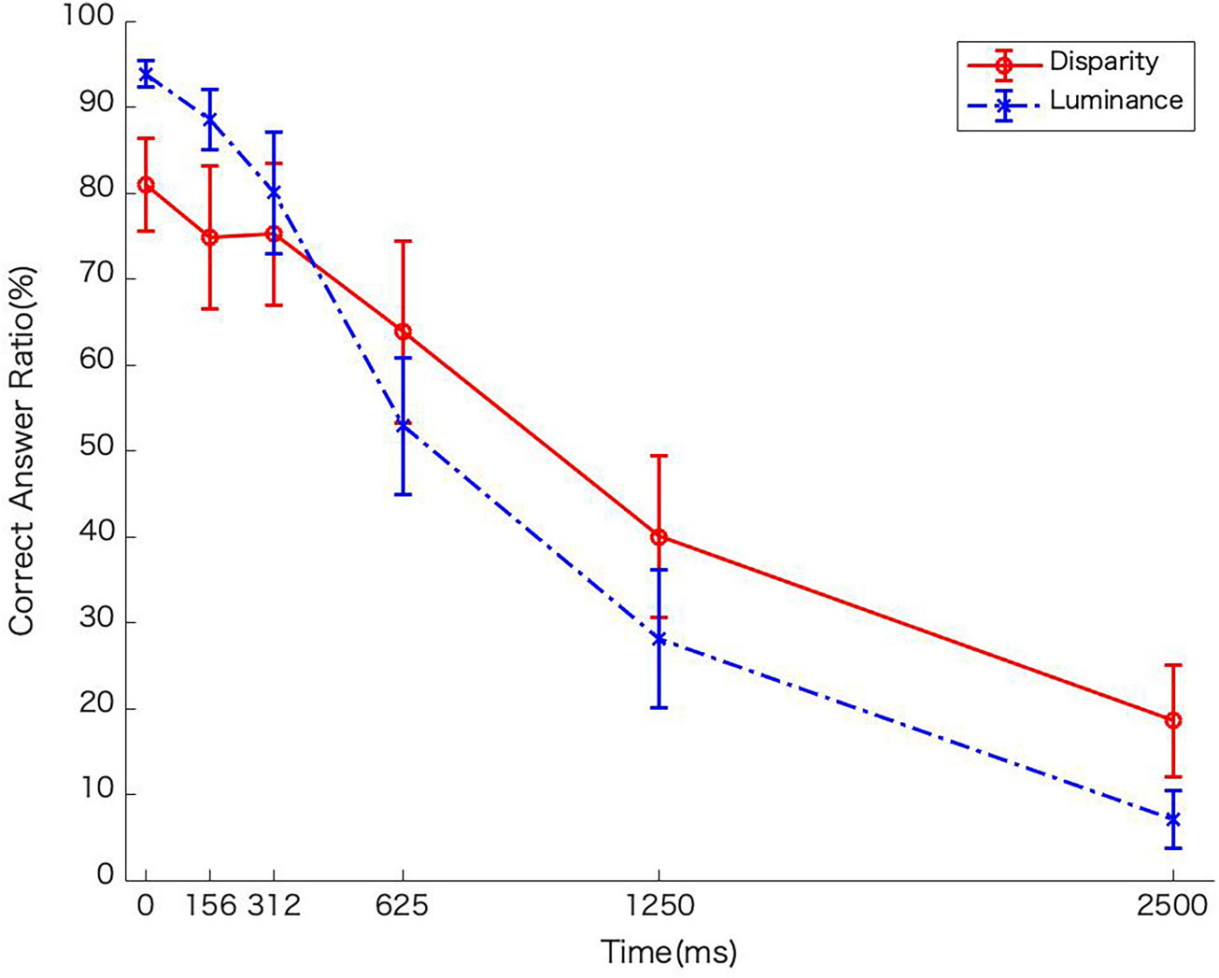
Average correct rate as a function of element onset asynchrony (EOA; ms) for two conditions of stimulus. The error bar shows the standard errors of the means. The horizontal axis shows the EOA (ms), the vertical axis shows the correct answer rate and the red and blue lines show the results of the binocular disparity condition and luminance condition, respectively.

Figure 3 shows the average rate of correct answers as a function of EOA for two conditions of the stimulus. The rate decreased as the EOA increased, with the luminance condition having a larger rate of decrease than the disparity condition. We conducted a two-way analysis of variance (ANOVA) of the stimulus type (luminance/disparity) and EOA that showed a significant effect for the EOA condition, *F*(5,30) = 87.642, *p *<.01, *η_G_*_2_ = .7060, but no main effect regarding the stimulus condition (luminance/disparity), *F*(1,6) = 0.006, *p *= .9408, *η_G_*_2_ <* *.001. The interaction of the factors was significant, *F*(5,30) = 5.33, *p *<* *.01, *η_G_*_2_ = .0947. Since the interaction was significant, a test for the simple main effect showed that the simple main effect of stimulus type was significant only for 2500 ms of EOA condition, *F*(1,6) = 7.75, *p *<* *.05, *η_G_*_2_ = .1702. The simple main effect of EOA was significant for all stimulus types, luminance: *F*(5,30) = 69.79, *p < *.001, *η_G_*_2_ = .834; and disparity: *F*(5,30) = 35.83, *p < *.001, *η_G_*_2_ = 0.5518. Based on these results, we conducted multiple comparisons among EOAs for each stimulus using Ryan's method. Under both disparity and luminance conditions, the rate of correct answers decreased as the EOA increased, but the effect of the EOA was smaller for the disparity stimulus compared to the luminance stimulus. Multiple comparisons showed a significant difference among all EOA values, except for the three shortest conditions (0, 156.25, and 312.5 ms) for the luminance stimulus (*p <* .05). However, in the disparity stimulus, there were significant differences except for the four shortest conditions (0, 156.25, 312.5, and 625 ms; *p <* .05).

### Discussion

In Experiment 1, the stimulus image defined by luminance and disparity was divided and presented with EOA as a variable, and the recognition rate of the number contained in the stimulus was obtained to verify the temporal integration characteristics of both disparity and luminance. The effect of the EOA was evident under both conditions, and it became difficult to integrate the information when the elements were presented over a long period. In addition, we identified some interaction between the EOA and the stimulus type (luminance/disparity). In the disparity condition, the rate of correct answers decreased more gradually than in the luminance condition as the EOA increased. This suggests that the binocular disparity is superior to luminance in the ability to integrate and restore sparsely presented information with EOA.

We assumed that the IMR would be important with respect to image perception. In previous studies in which a drawn image was divided and presented spatiotemporally as in the present experiment, all elements were manipulated to be presented for an equal duration (Ikeda & Uchikawa, 1978; Unuma, 1992). In Experiment 1 of the current study, all of the divided elements were displayed for the same duration, as in the previous studies. In this case, the trend in the results may be due to the IMR, as opposed to the EOA, among elements. In addition, it is possible that the rate of correct answers was high because the IMR was large, as opposed to a short EOA. In Experiments 2 and 3, we kept the IMR constant and investigated the temporal integration characteristics of luminance and disparity for image perception.

## Experiment 2: Effect of EOA and Presentation Order on Temporal Integration of Shape Recognition Under Constant IMR

In Experiment 1, the temporal integration was compared using stimuli defined by luminance and binocular disparity. However, the IMR changed for each EOA condition in Experiment 1. In Experiment 2, we conducted the same experiment as in Experiment 1, with the IMR being constant among the conditions. If the property of shape recognition were to vary with EOA, then the correct response rate would decrease as EOA increases by lower-order temporal integration. On the other hand, if IMR is more important for shape recognition, then the correct response rate would not change with EOA but would change with IMR conditions.

In addition, to investigate the effect of motion information on the integration of disparity and luminance integration in shape recognition, we added the condition of slit vision, in which the visible area moved gradually from the edge of the target to the opposite side. If the correct response rate increases with this slit viewing condition, we can assume the global motion information contributes to shape recognition by spatiotemporal integration along the motion trajectory of the slit, indicating that higher-order spatiotemporal integration occurs for visual object recognition.

### Methods

#### Participants

Seven participants, including the three authors, who also participated in Experiment 1, participated in Experiment 2.

#### Stimuli

There were four conditions of stimulus presentation: stimulus type (luminance/disparity), EOA from the onset of the first element to the onset of the last element (156.25, 312.5, 625, 1250, and 2500 ms), presentation order (random/slit vision), and IMR (12%/24%).

**Figure 4.**
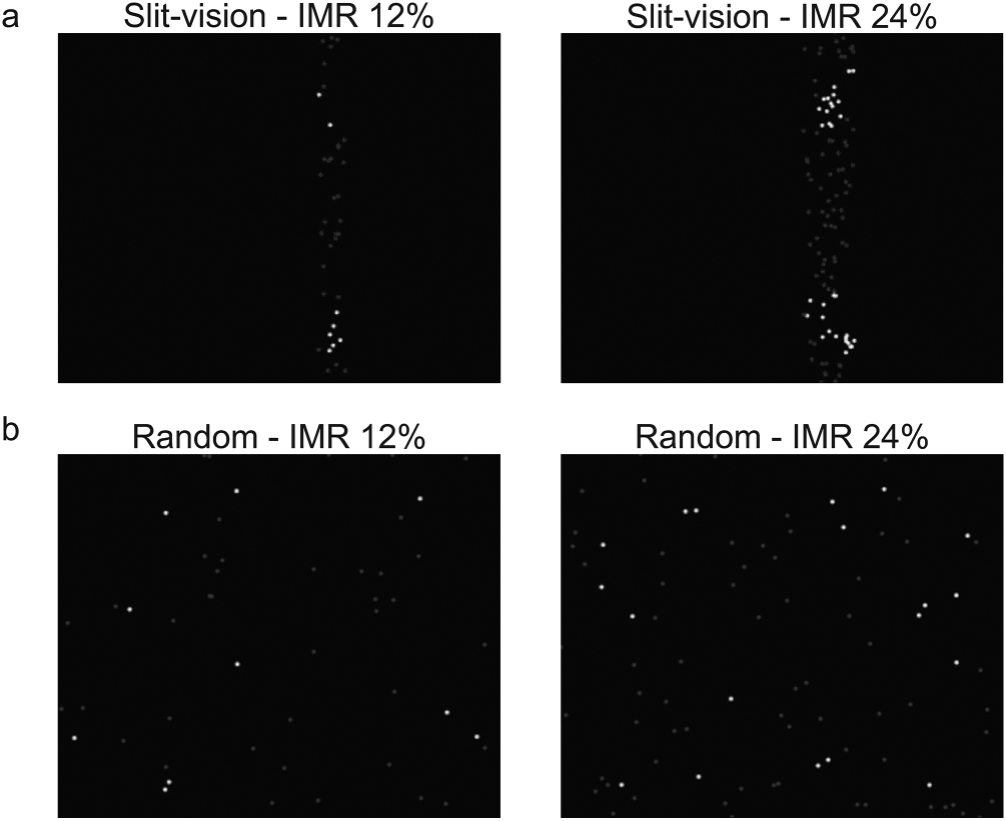
(a) The stimulus image in the instantaneous maximum ratio (IMR) is 12% (left) and 24% (right) condition in slit-vision. (b) The stimulus image in the IMR 12% (left) and 24% (right) condition in random presentation.

Under slit-vision conditions, the stationary stimulus was viewed through a vertical slit window moving from right to left at a constant speed (57.28°/s, 28.64°/s, 14.32°/s, 7.16°/s, and 3.58°/s; Figure 4(a)). Under the random presentation, the element dots were presented in random order (Figure 4(b)). The number of dots presented per frame (16.67 ms) and the presentation duration of each dot were set to be equal between these two conditions.

There were two conditions for the IMR: 12% and 24%. The instantaneous maximum number for the 12% IMR condition was 96, and a total of 800 dots were presented on the screen. The instantaneous maximum number for the 24% IMR condition was 192 (Figure 4). Under the slit-vision condition, the width of the slit changed due to the IMR. The slit width was 36 pixels (1.08°) for 12% IMR and 72 pixels (2.16°) for 24% IMR.

**Figure 5.**
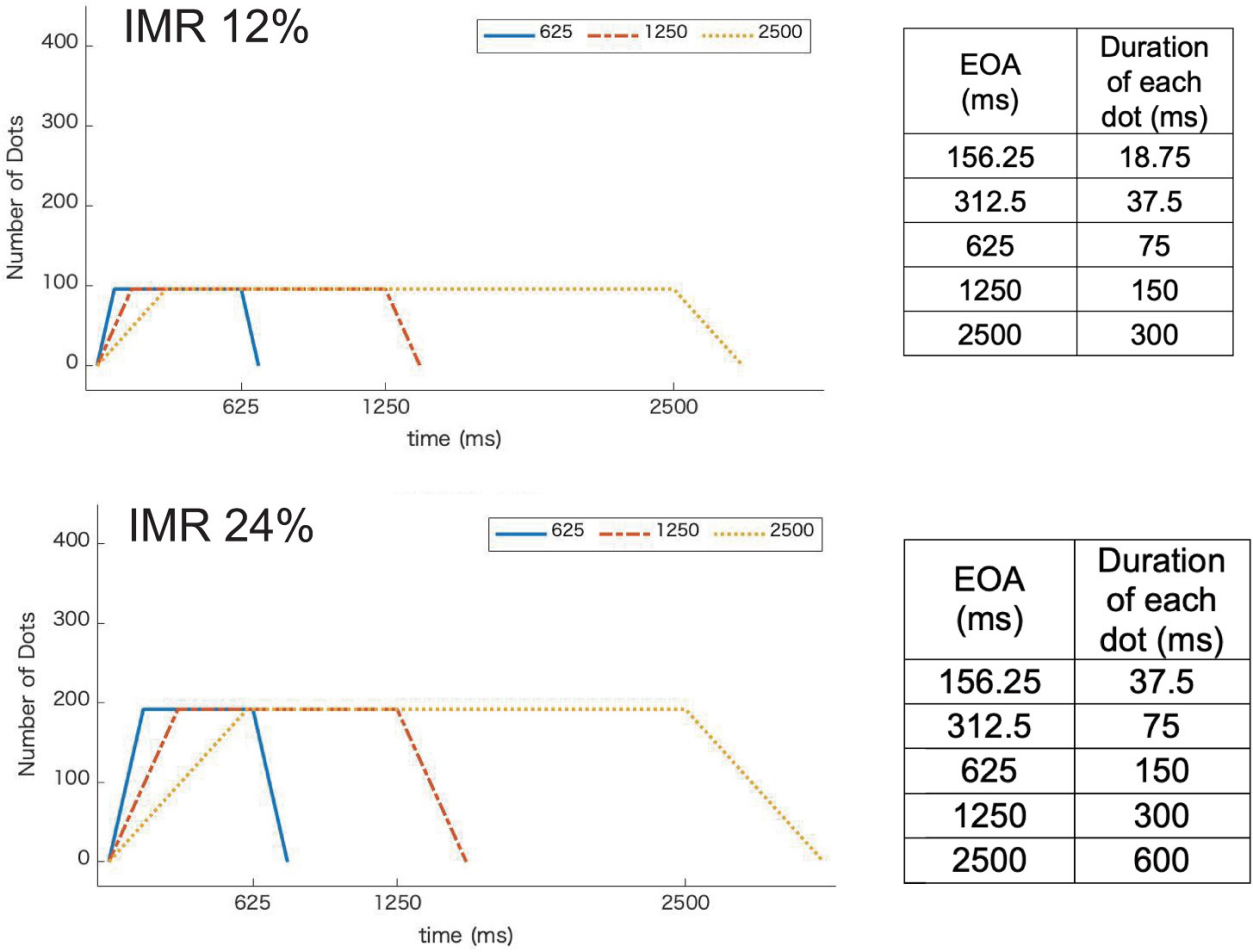
Time course of the number of presented dots in three EOA conditions (625, 1250, and 2500 ms). The upper figure shows the conditions for the IMR 12%. The lower figure shows the conditions for the IMR 24%. The horizontal axis is time (ms), and the vertical axis is the number of presented dots. The right tables show the duration of one dot for each EOA.

*Note*. EOA = element onset asynchrony; IMR = instantaneous maximum ratio.

The presentation duration of each dot differed depending on the combination of the IMR and EOA conditions. The tables in Figure 5 show the presentation duration of each dot for each IMR. In Experiment 2, the shorter the EOA, the shorter the presentation duration of each dot. Figure 5 shows the time course of the number of dots displayed for the three EOA conditions (625, 1250, and 2500 ms). In Experiment 1, the number of dots presented at the same time differed, depending on the EOA condition (Figure 2); however, in Experiment 2, the number of dots was fixed for each IMR condition.

#### Procedures

The procedure was the same as in Experiment 1. The participant responded with a two-digit number using the numerical keypad. Under the slit-vision condition, the stimulus elements for the vertical slit were presented from right to left, and the participants were allowed to move their eyes to pursue the slit during the presentation. Each participant responded with a total of 800 trials (stimulus type 2 × presentation order 2 × IMR 2 × EOA 5 × 20 repetitions). Forty combinations of the condition were presented in random order in a block, and the block was repeated 20 times.

### Results

**Figure 6.**
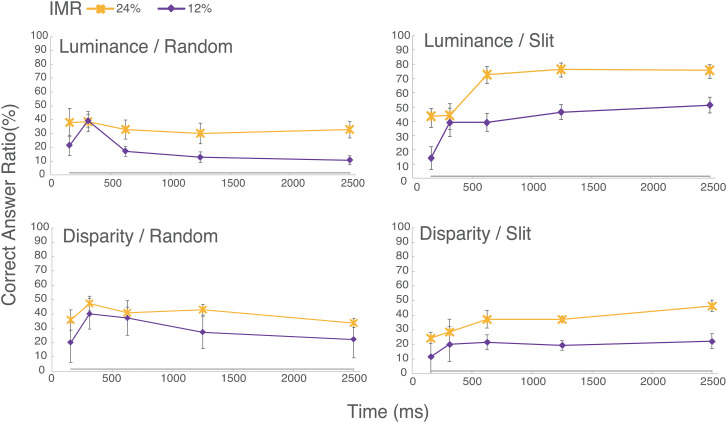
Averaged correct rate across participants as a function of EOA. The error bars show the standard errors of the means. The horizontal axis is the EOA (ms) and the vertical axis is the correct answer rate. The left and right panels show the results of random and slit-vision conditions, respectively. The upper and lower panels show the results of luminance and disparity stimuli, respectively. The symbol indicates the condition of IMR.

*Note*. EOA = element onset asynchrony; IMR = instantaneous maximum ratio.

The left side of Figure 6 shows the results of the correct response rate when the dots were presented in a random presentation order. First, in the random viewing condition, both shape recognition by luminance and by disparity showed a slight increase in correct response rate with shorter EOA, peaking at 300 ms. The correct response rate was higher for the 24% than for the 12%.

The right-hand side of Figure 6 shows the average correct response rate for the slit viewing conditions for the luminance and disparity stimuli. In the luminance condition, the correct response rate clearly improved as the EOA increased. In the disparity condition, the trend was less clear than in the luminance condition, but there was a slight improvement in the correct response rate with the EOA. Furthermore, as in the random viewing condition, the difference in correct response rate by IMR was obvious for all EOAs, with 24% IMR having a higher correct response rate.

The result shows that the correct response rate improves when the IMR is high in both the random and slit viewing conditions. Furthermore, in the random viewing condition, the correct response rate tended to improve with shorter EOA, while in the slit viewing condition, the correct response rate tended to improve with longer EOA, that is, with slower slit window motion speed. To examine this trend statistically, we performed a four-way ANOVA (stimulus type, presentation order, IMR, and EOA) and identified IMR and EOA as significant effects, IMR: *F*(1,6) = 100.42, *p *<* *.01, *η_G_*_2_ = .1179; EOA: *F*(4,24) = 5.554, *p* <* *.01, *η_G_*_2_ = .0370. In addition, there were significant interactions between the stimulus type and presentation order, *F*(1,6) = 93.687, *p *<* *.01, *η_G_*_2_ = .1071, and between the EOA and presentation order, *F*(4,24) = 18.062, *p *< .01, *η_G_*_2_ = .0730. Furthermore, the second-order interaction of stimulus type, presentation order, and EOA was significant, *F*(4,24) = 6.67, *p < *.001, *η_G_*_2_ = .023. We found simple interaction effect of stimulus type × presentation condition at EOAs 312.5 ms: *F*(1,6) = 12.36, *p *< .05, *η_G_*_2_ = .0477; 625 ms: *F*(1,6) = 51.23, *p *< .001, *η_G_*_2_ = .1635; 1250 ms: *F*(1,6) = 119.17, *p *< .001, *η_G_*_2_ = .2453; and 2500 ms: *F*(1,6) = 20.15, *p *< .01, *η_G_*_2_ = .1524; simple interaction effect of stimulus type × EOA for both slit, *F*(4,24) = 2.79, *p *< .05, *η_G_*_2_ = .027, and random presentation, *F*(4,24) = 3.12, *p *< .05, *η_G_*_2_ = .0214; simple interaction effect of presentation order × EOA for both luminance, *F*(4,24) = 26.97, *p *< .001, *η_G_*_2_ = .205, and disparity stimuli, *F*(4,24) = 3.70, *p *< .05, *η_G_*_2_ = .0251.

We found the simple–simple main effect of stimulus type on the slit presentation at the last three EOAs, 625 ms: *F*(1,6) = 11.39, *p < *.05, *η_G_*_2_ = .0238; 1250 ms: *F*(1,6) = 13.85, *p < *.01, *η_G_*_2_ = .368; 2500 ms: *F*(1,6) = 7.45, *p <*0.05, *η_G_*_2_ = .0247, but no simple–simple main effect of stimulus type on the random presentation at any EOAs. Interestingly, also we found the simple–simple main effect of presentation order on the luminance stimulus at the last three EOAs, 625 ms: *F*(1,6) = 72.3, *p < *.001, *η_G_*_2_ = 0.4302; 1250 ms: *F*(1,6) = 122.19, *p < *.001, *η_G_*_2_ = .629; 2500 ms: *F*(1,6) = 63.28, *p <*0.001, *η_G_*_2_ = 0.6681, and the simple–simple main effect of presentation order on the disparity stimulus at the first two EOAs, 156.25 ms: *F*(1,6) = 6.46, *p < *.05, *η_G_*_2_ = .0426; 312.5 ms: *F*(1,6) = 8.76, *p < *.05, *η_G_*_2_ = .1074. This simple–simple main effect indicates the importance of each element being presented at short time intervals in the random local presentation of images especially for disparity stimulus, on the other hand, the significance of a longer presentation for each element in the spatiotemporal integration of global motion through slit vision for luminance stimulus. Finally, we found the simple–simple main effect of EOAs on all combinations except Disparity × Slit (Luminance × Slit): *F*(4, 24) = 11.5, *p < *.001, *η_G_*_2_ = 0.3284; Luminance × Random: *F*(4,24) = 7.16, *p < *.001, *η_G_*_2_ = 0.1244; Disparity × Random: *F*(4,24) = 4.90, *p < *.01, *η_G_*_2_ = .0706.

From the ANOVA results, we conducted multiple comparisons for the EOA condition with Ryan's method. Under the luminance and slit viewing conditions (the upper right panel in Figure 6), there was a significant difference (*p <* .05) between EOA 156 ms and other EOAs and between EOA 312 ms and other EOAs. Namely, the percentage of correct responses increased significantly as EOA increased. Under the luminance and random viewing conditions (the upper left panel in Figure 6), there was a significant difference (*p <* .05) between EOA 312 ms and EOA 625 ms and beyond, confirming a peak at EOA 312 ms. In the disparity and random viewing condition (the lower left panel in Figure 6), significant differences were found between EOA 156 ms and 312 ms and between EOA 312 ms and 2500 ms (*p <* .05), confirming a gradually increasing trend due to EOA statistically. We did not find the simple–simple main effect of EOA, but we found significant differences were found only between EOA 156 ms and EOA 2500 ms (*p <* .05) in the disparity and slit viewing condition (the bottom right panel in Figure 6).

### Discussion

One of the purposes of Experiment 2 was to separate the influence of the IMR from the temporal integration characteristics. If low-order temporal integration is important in shape recognition, the correct response rate would decrease with increasing EOA. On the other hand, if the presentation duration of each element is important in shape recognition, the correct response rate should increase as EOA increases, since the presentation time of each element and EOA covary. Another prediction is that if the number of dots presented at the same time is important, then there should be a difference in the correct response rate between the two IMR conditions.

First, the results consistently showed that the rate of correct response was high when the IMR was large under all conditions, suggesting that IMR may be the main factor in the improvement in shape recognition with the short EOA as in Experiment 1. This indicates that, if a large portion of the stimulus elements are seen at a certain moment, then the shape recognition performance improves, which is a reasonable result. Within this effect, in the 12% IMR condition, the correct response rate had a significant peak at EOA 312 ms for both luminance and disparity stimuli. It may be that the shape recognition system of the visual system actively attempts to perform temporal summation in low-information situations.

Another purpose of this experiment was to investigate the effect of the spatial order of element presentation. For this purpose, we added a slit-view condition where observing the image of a stationary object through a slit moving from right to left. As with random viewing, a robust effect of IMR was observed in the slit viewing. We found an interaction between the presentation order and the stimulus type (disparity/luminance), indicating the difference in the slit vision on the temporal integration of disparity and luminance. Furthermore, as a result, specific to slit viewing, the longer the EOA in the luminance stimulus condition, that is, the slower the slit window speed, the higher the correct response rate. The effect of improving the recognition accuracy in slit vision is especially remarkable to the luminance stimulus, and it can be inferred that this effect is small in the disparity stimulus. This indicates that the spatiotemporal integration process using luminance motion information contributes to the processing of shape recognition, rather than simply low-order temporal summation within a short period of presentation. These results indicate that the temporal integration mechanism in localized areas is common to disparity and luminance, but that for global motion shows differences between the two mechanisms. We will discuss about the possibility in the General discussion section.

One of the possible causes of the lack of an increase in the correct response with slit presentation for the disparity stimulus would be the effect of eye movements. Because the participant could move their eyes during observation, the pursuit eye movement may be induced by the slit motion. In the case of random presentation, the participant always observed the center of the stimulus, because they could not predict which elements were shown in the next phase. On the other hand, in the case of slit vision, it is considered that the participant moves the eyeball from right to left, along with the movement of the slit. With a stereo stimulus, theoretically, diplopia would occur unless the corresponding points of the left and right retinal images matched exactly, which makes it difficult to obtain binocular disparity information or allows for only degraded disparity information to be acquired (Otero-Millan et al., 2014). If participants moved their eyes, the position of the images would be blurred, which makes it difficult to match the two images accurately; in this case, the rate of correct answers may not improve only in the slit-vision condition for the disparity. In fact, some participants reported diplopia due to difficulties in binocular fusion during the slit-vision presentation. The latency of vergence eye movements required for stereopsis is approximately 170 ms (Yang et al., 2002), and it is possible that following the moving slit may interfere with the vergence eye movements, resulting in that participants could not establish stereoscopic vision. Therefore, in Experiment 3, to investigate the effect of eye movements, we added a condition in which the stimulus moved behind a static slit and no eye movements would occur.

## Experiment 3: Temporal Integration of Binocular or Luminance Cues for Shape Recognition of a Moving Object Behind a Stationary Slit

Experiment 2 showed that the rate of correct answers in the slit-vision condition increased compared to that of the random presentation for the luminance stimulus; however, no difference was observed for the disparity stimulus. It is possible that disparity detection ability may degrade due to eye movement following the moving slit. In Experiment 3, the position of the slit window was fixed, and the stimulus was presented to move behind the slit. In this case, the IMR was unchanged, but the eye movement did not pursue the slit. If the shape recognition by the binocular disparity was degraded due to the pursuit eye movement in Experiment 2, the presentation order should not affect the rate of correct answers in Experiment 3.

### Methods

#### Participants

Seven people, the same individuals who participated in Experiments 1 and 2, also participated in Experiment 3.

#### Stimuli

The stimuli were the same as in Experiments 1 and 2. In Experiment 3, the slit window was fixed to the screen and the stimulus dot moved from right to left behind the slit window. The participant could observe the whole image of the stimulus by keeping a steady gaze on the center of the screen. Similar to Experiment 2, there were three conditions of stimulus presentation: stimulus type (luminance/disparity), IMR (12%/24%), and EOA (156.25, 312.5, 625, 1250, and 2500 ms). The size of the slit window differed depending on the IMR (12%: 1.08°; 24%: 2.16°), and the dot speed differed depending on the condition of EOA (57.28°/s, 28.64°/s, 14.32°/s, 7.16°/s, and 3.58°/s). These speeds were equal to the speed of the moving slit in Experiment 2.

#### Procedures

The experimental procedure was the same as in Experiment 2. Each participant responded, with a total of 400 trials (stimulus type 2 × IMR 2 × EOA 5 × 20 repetitions).

### Results

**Figure 7.**
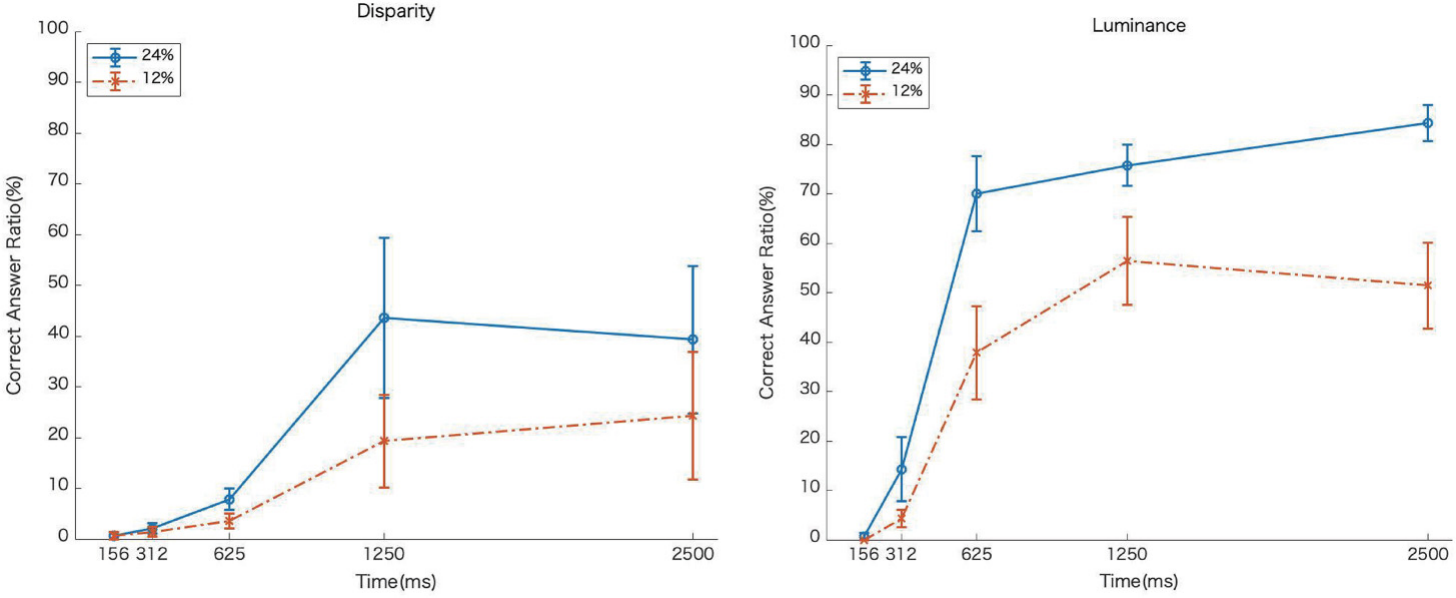
Averaged correct rate as a function of element onset asynchrony (EOA) in Experiment 3. Error bars show the standard error of the means. The horizontal axis shows the EOA (ms) and the vertical axis shows the correct answer rate. The left and right panels show the results of disparity and luminance conditions, respectively.

**Figure 8.**
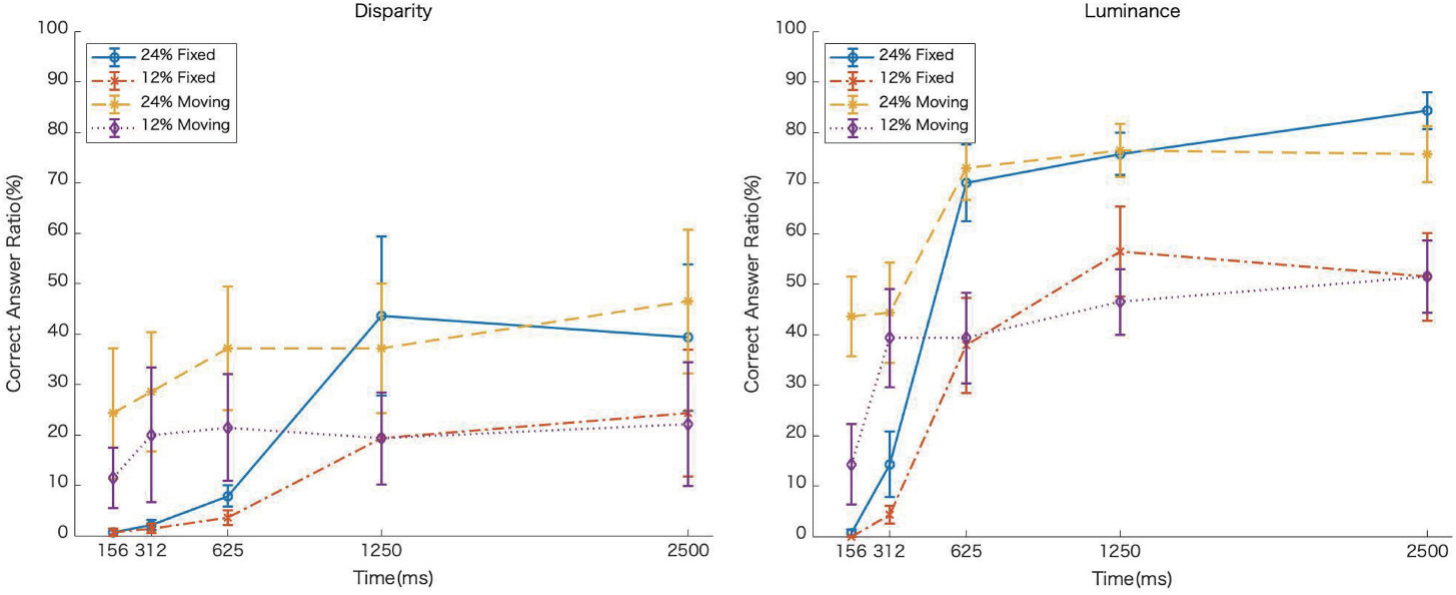
Comparison of the results in Experiments 2 and 3. The dotted and solid lines indicate the results of Experiments 2 and 3, respectively. The left and right panels show the results of disparity and luminance conditions, respectively.

Figure 7 shows the average rate of correct answers as a function of EOA, and Figure 8 shows a graph comparing the results of the moving slit in Experiment 2. For all of the conditions, the longer the EOA, the higher the rate of correct answers. Under the fixed-slit condition, the rate of correct answers was lower when the EOA was short, compared to the moving-slit condition (Figure 8). However, when the EOA was long, there were no differences between the moving and fixed-slit conditions. Furthermore, whereas the correct response rate was not so much different among EOAs in disparity condition in Experiment 2, the correct rate increased with EOAs in Experiment 3, indicating that the correct rate for disparity-defined image recognition was improved by the global motion of image behind the fixed slit.

Four-way ANOVA was conducted on these data for the factors of stimulus type (disparity or luminance), slit type (fixed or moving slit), IMR, and EOA. The results showed significant main effects for all factors, other than slit type, stimulus type: *F*(1,6) = 21.326, *p *<* *.01, *η_G_*_2_ = .2583; IMR: *F*(1,6) = 68.505, *p *< .01, *η_G_*_2_ = .1452; and EOA: *F*(4,24) = 44.515, *p* <* *.01, *η_G_*_2_ = .3398. No significant effect was found for the main effect of slit type, *F*(1,6) = 5.881, *p* = .0515, *η_G_*_2_ = .0739. The interaction between the slit type and IMR, *F*(1,6) = 18.137, *p *< .01, *η_G_*_2_ = .0056, and that between the slit type and EOA, *F*(4,24) = 7.154, *p* < .01, *η_G_*_2_*_ _* = .0835, were also significant. We found the second-order interactions of stimulus type × IMR × EOA: *F*(4,24) = 3.67, *p* < .05, *η_G_*_2_*_ _* = .007; stimulus type × slit movement × EOA: *F*(4,24) = 3.20, *p* < .05, *η_G_*_2_*_ _* = .0193; IMR × slit movement × EOA: *F*(4,24) = 2.80, *p* < .05, *η_G_*_2_*_ _* = 0.007. Now we focus on the simple–simple effect of slit movement on the stimulus type × EOA interaction. There were significant simple–simple effects of slit movement on the luminance stimulus at 156.25  ms: *F*(1,6) = 14.00, *p* < .01, *η_G_*_2_*_ _* = .5198; and 312.5 ms: *F*(1,6) = 14.03, *p* < .01, *η_G_*_2_*_ _* = .4242. It means that under the conditions of a long EOA, the type of slit, fixed or moving, does not affect the rate of correct answers for luminance stimulus. In other words, these significant simple main effects of the slit viewing indicate that the rise of correct rate by temporal integration in the shorter EOAs, as seen during moving-slit observation in Experiment 2, is suppressed in fixed-slit viewing.

### Discussion

In Experiment 3, we investigated whether the results of Experiment 2 that the rate of correct answers did not increase in the slit-vision condition for the disparity stimulus, was due to the eye movements pursuing the moving slit. If this assumption is valid, the rate of correct answers should be higher under the fixed-slit condition than in the moving-slit condition. There was a significant simple–simple effect of slit type on luminance stimulus at short EOAs. Under the long-EOA condition, there were no differences in the rate of correct answers, regardless of the slit type and stimulus type. Furthermore, the effect of temporal integration at short EOAs that was seen in Experiment 2 also disappeared in the fixation slit condition. However, there was also no improvement in the correct response rate for the long EOAs due to restricted eye movements.

We consider that it is likely that the disappearance of the afterimage and the speed of the stimuli induce the low rate of correct answers for fixed-slit conditions with a short EOA. After seeing a strong light, participants perceive the afterimage for some time after the light disappears (Cohen-Duwek & Spitzer, 2018). Under the moving-slit condition, each dot was presented at a different position on the display. Therefore, it is possible that the afterimage may be perceived under the short EOA condition in which the slit moves at a very high speed and the participants show only a slight eye movement in response. In this case, the dots can be perceived for a longer time than the actual presentation time, even for a short presentation. On the other hand, under the fixed-slit condition, the shorter the presentation time, the faster the stimulus movement. Therefore, all of the dots were presented within a narrow spatial area in the slit, and the subsequent dot group masked the afterimage. It is considered that the participant did not perceive any afterimage and that the information for recognition was not sufficient for short EOA.

Although the variance was large and no simple–simple effect of slit type was observed, the correct response rate decreased in the fixed-slit condition at short EOAs even in the disparity condition. A possible reason could be due to the loss of eye movement-derived integration, which was controlled in Experiment 3. For example, Souto et al. (2019) investigated thresholds for coherent motion during pursuit eye movements and reported reduced sensitivity to coherent motion in the direction opposite to the pursuit eye movement. This indicates that somatic motor information or efference copy information during pursuit eye movements plays a more important role with respect to visual field stability and motion detection according to pursuit eye movements. In addition to the local spatial masking, the lack of eye movement information in our experiments may have made spatiotemporal integration more difficult in the short EOA.

## General Discussion

We intended to clarify the recognition mechanism based on the temporal integration of binocular disparity by comparing that of luminance. In Experiment 1, two-digit numbers defined by random-dot stereograms by luminance were presented with EOA. The results showed that the rate of correct answers was high when the EOA was short under both disparity and luminance stimuli. In addition, the rate in the disparity condition decreased more gradually than in the luminance condition with an increase in the EOA, suggesting that binocular disparity is superior to luminance in the ability to integrate and restore sparsely presented information. In Experiments 2 and 3, when the ratio of observable dots (IMR) was fixed, quick stimulus presentation maximized the correct answer ratio under random conditions, suggesting rapid shape recognition is possible for the pattern even with binocular disparity. Slit vision with a longer exposure (EOA) increased the correct answer ratio for luminance-defined stimuli, implying spatiotemporal integration for shape recognition. The properties of temporal integration were similar for luminance and disparity, indicating there is a common time limit for shape recognition from distributed luminance and disparity data. However, luminance stimuli in slit vision yielded higher accuracy than random vision, unlike disparity stimuli.

Our results suggest that IMR may be important in shape recognition when local information is presented sporadically. In previous studies such as Ikeda and Uchikawa (1978), where images were segmented and presented in a random order, the segmented parts retained local information. In our study, since random dots were used, little local spatial information was preserved in the random viewing condition. In the slit viewing condition, little local spatial information was also retained either. The results of Experiments 1 and 2 indicate that the amount of spatial information presented at the same time for the whole is an important factor for shape restoration, regardless of whether luminance- or disparity-defined stimuli.

Furthermore, it was newly revealed that given coherent motion information of the slit window, the spatiotemporal integration of disparity information was less conducive to shape recognition processing than that of luminance information. Comparing the slit viewing conditions in Experiment 2 for the luminance and disparity stimuli, we found that when the slit velocity slowed down for the luminance stimulus, more information integration occurred and it improved the correct response rate, but not for the disparity stimulus. We hypothesized that this might be because pursuit eye movements to the slit window suppressed some important elements of binocular vision, such as vergence eye movements. In Experiment 3, we used a fixed-slit window to prevent pursuit eye movements. In both the disparity and luminance conditions, the overall correct response rate did not improve, and the correct response rate at the short EOA was lower in the fixed-slit condition than in the moving-slit condition. Those results indicate that the suppression of retinal slip by the pursuit of eye movements is effective in low-order temporal integration. Our assumption here is that the low-order temporal integration is similar to the summation of local spatial information in the temporal impulse response seen in luminance and binocular disparity (Cox et al., 2019). Such lower-order temporal integration would require a clearer retinal image input. If the temporal integration of short EOAs was suppressed by retinal slips of moving images in Experiment 3, we suppose that the results of the disparity stimuli obtained in Experiment 2, in which the correct response rate seems to be flat for EOA duration, would be a result of a combination of an effect of low-order temporal integration and an effect of high-order spatiotemporal integration by coherent motion information. If this interpretation is correct, then the contribution of spatiotemporal integration of disparity information for shape perception may be less than that of luminance information. But we suppose the contribution is not completely absent, because some previous studies of the Gestalt school of psychology have reported that shape recognition of lemon-like objects defined by binocular disparity through a moving-slit window was possible for many observers, although no objective data were presented (Fujita, 1990). Future research would require experiments to separate the effects of temporal integration with short EOA from those of spatiotemporal integration with global motion information.

Motion information is used for the reconstruction of divided images of the stimulus defined by luminance for perceiving the global image, suggesting the contribution of the output of middle temporal (MT) pattern cells to image recognition (Nishida, 2004). The random presentation and slit-vision condition used in the present study differed not only in the order of dot presentation, but also in whether they included the motion of the presented area of the stimulus. In the slit-vision condition of the luminance stimulus, global integration may have occurred due to the contribution of the MT field neurons. On the other hand, we did not find such a contribution of the motion information in the integration for the disparity stimulus. This suggests that such higher-order motor mechanisms are not involved in the process of binocular disparity integration for pattern perception.

Although it is possible to detect local one-dimensional motion in V1, the process of integrating local motion information into a global motion is beyond the MT area (Rust et al., 2006). It has also been reported that when different patterns of motion are presented to the left and right eyes, the information is not integrated into a global motion (Tailby et al., 2010). These observations indicate that the perception of global motion is mostly processed in MT, but some of the information for perceiving global motion must be expressed by the monocular neurons in V1. Our result that the performance for perceiving global image from disparity did not increase in slit vision compared to the random presentations is consistent with previous studies in the sense that the combined information of both eyes is not used for perceiving global motion.

In this study, we created stimuli defined by luminance and disparity and examined their characteristics of spatiotemporal integration for perceiving global shapes. We found that global motion information contributes to the perception of global shape only for a luminance-defined pattern not for a disparity-defined pattern. The two temporal characteristics would likely complement each other, and stimuli that combine disparity and luminance would have an advantage for shape recognition. Thus, as a next step, we plan to investigate the characteristics of spatiotemporal integration for stimuli that use both luminance and disparity. We hypothesize that the combination of these two cues will improve the ability to perceive the global image.

## Supplemental Material

sj-gif-1-ipe-10.1177_20416695231224138 - Supplemental material for Temporal integration characteristics of an image defined by binocular disparity cues Click here for additional data file. Supplemental material, sj-gif-1-ipe-10.1177_20416695231224138 for Temporal integration characteristics of an image defined by binocular disparity cues by Fumiya Haraguchi, Rumi Hisakata and Hirohiko Kaneko in i-Perception

sj-gif-2-ipe-10.1177_20416695231224138 - Supplemental material for Temporal integration characteristics of an image defined by binocular disparity cues Click here for additional data file. Supplemental material, sj-gif-2-ipe-10.1177_20416695231224138 for Temporal integration characteristics of an image defined by binocular disparity cues by Fumiya Haraguchi, Rumi Hisakata and Hirohiko Kaneko in i-Perception

sj-gif-3-ipe-10.1177_20416695231224138 - Supplemental material for Temporal integration characteristics of an image defined by binocular disparity cues Click here for additional data file. Supplemental material, sj-gif-3-ipe-10.1177_20416695231224138 for Temporal integration characteristics of an image defined by binocular disparity cues by Fumiya Haraguchi, Rumi Hisakata and Hirohiko Kaneko in i-Perception
