# Supplementary figures and images for: Temporal integration characteristics of an image defined by binocular disparity cues
Source: Iperception. 2024 Jan 9;15(1):20416695231224138. doi: 10.1177/20416695231224138 (PMC10777792; doi:10.1177/20416695231224138)

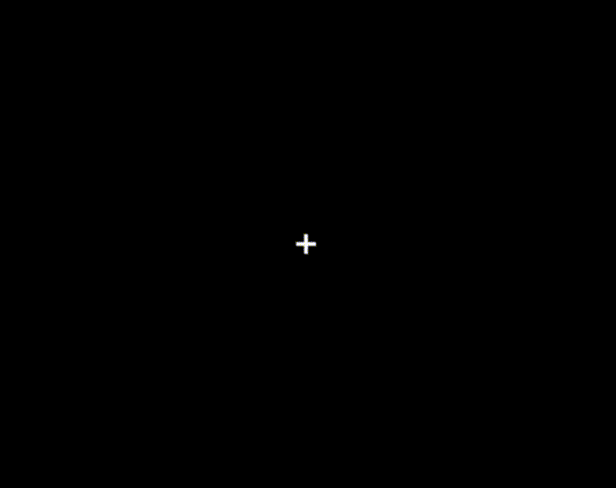

Supplement: sj-gif-1-ipe-10.1177_20416695231224138 - Supplemental material for Temporal integration characteristics of an image defined by binocular disparity cues [file sj-gif-1-ipe-10.1177_20416695231224138.gif]

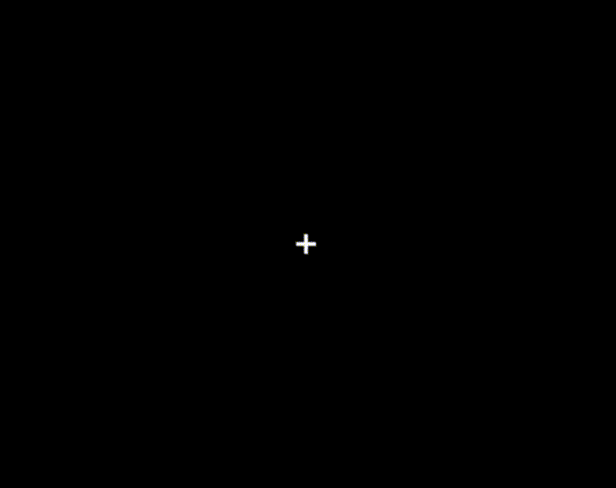

Supplement: sj-gif-2-ipe-10.1177_20416695231224138 - Supplemental material for Temporal integration characteristics of an image defined by binocular disparity cues [file sj-gif-2-ipe-10.1177_20416695231224138.gif]

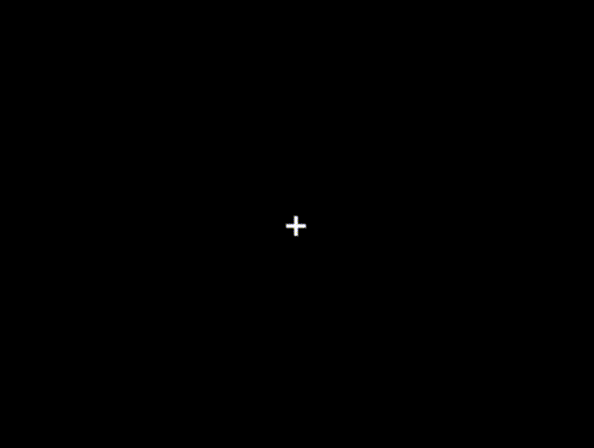

Supplement: sj-gif-3-ipe-10.1177_20416695231224138 - Supplemental material for Temporal integration characteristics of an image defined by binocular disparity cues [file sj-gif-3-ipe-10.1177_20416695231224138.gif]
